# Axonal transport in a peripheral diabetic neuropathy model: sex-dimorphic features

**DOI:** 10.1186/s13293-018-0164-z

**Published:** 2018-01-19

**Authors:** Marzia Pesaresi, Silvia Giatti, Roberto Spezzano, Simone Romano, Silvia Diviccaro, Tiziana Borsello, Nico Mitro, Donatella Caruso, Luis Miguel Garcia-Segura, Roberto Cosimo Melcangi

**Affiliations:** 1 Dipartimento di Scienze Farmacologiche e Biomolecolari, Università degli Studi di Milano, Milano, Italy; 2 Department of Neuroscience, IRCCS-Mario Negri Institute for Pharmacological Research, Milano, Italy; 3 Instituto Cajal, CSIC, CIBER de Investigación Biomédica en Red de Fragilidad y Envejecimiento Saludable (CIBERFES), Instituto de Salud Carlos III, Madrid, Spain

**Keywords:** Streptozotocin, Sciatic nerve, Dorsal root ganglia, Mitochondria, Neuroactive steroids, Male, Female

## Abstract

**Background:**

Disruption of axonal transport plays a pivotal role in diabetic neuropathy. A sex-dimorphism exists in the incidence and symptomatology of diabetic neuropathy; however, no studies so far have addressed sex differences in axonal motor proteins expression in early diabetes as well as the possible involvement of neuroactive steroids. Interestingly, recent data point to a role for mitochondria in the sexual dimorphism of neurodegenerative diseases. Mitochondria have a fundamental role in axonal transport by producing the motors’ energy source, ATP. Moreover, neuroactive steroids can also regulate mitochondrial function.

**Methods:**

Here, we investigated the impact of short-term diabetes in the peripheral nervous system of male and female rats on key motor proteins important for axonal transport, mitochondrial function, and neuroactive steroids levels.

**Results:**

We show that short-term diabetes alters mRNA levels and axoplasm protein contents of kinesin family member KIF1A, KIF5B, KIF5A and Myosin Va in male but not in female rats. Similarly, the expression of peroxisome proliferator-activated receptor γ co-activator-1α, a subunit of the respiratory chain complex IV, ATP levels and the key regulators of mitochondrial dynamics were affected in males but not in females. Concomitant analysis of neuroactive steroid levels in sciatic nerve showed an alteration of testosterone, dihydrotestosterone, and allopregnanolone in diabetic males, whereas no changes were observed in female rats.

**Conclusions:**

These findings suggest that sex-specific decrease in neuroactive steroid levels in male diabetic animals may cause an alteration in their mitochondrial function that in turn might impact in axonal transport, contributing to the sex difference observed in diabetic neuropathy.

## Background

Peripheral diabetic neuropathy (PDN) is one of the most prevalent complications of diabetes [1]. It can take different forms, the most common of which is the length-dependent axonal sensorimotor and autonomic neuropathy. This is associated with structural changes in the peripheral nerves, degeneration and impaired regeneration ability due to a dying-back of distal axons.

Hyperglycemia, a key factor in the pathogenesis of diabetic complications, mediates phenotypic changes in mitochondria resulting in increased ROS production, ATP depletion, and altered calcium homeostasis [2]. The outcome is the exhaustion of the ATP supply for energy consuming processes in neurons such as axonal transport. This process is fundamental to maintain neuronal homeostasis, especially in the peripheral nervous system (PNS) in which long axons constitute a major challenge for the movement of cargoes. Cargoes are predominantly transported along microtubules via ATP-dependent motor proteins. The kinesin superfamily of proteins and cytoplasmic dynein are the main microtubule-based motor proteins [3]. In addition, cargoes can also be transported bidirectionally for short distances along actin filaments via myosin motors [4].

Growing evidence indicates that axonal transport is impaired in diabetes, probably contributing to the development of neurological complications and subsequently diabetic neuropathy [5–9]. However, despite the main role of molecular motors in this process, a limited amount of work has been performed to analyze the effect of diabetes on local changes in motor proteins in PNS [7, 9]. Moreover, to our knowledge, no studies have been performed to analyze sex differences in their levels and expression under diabetic conditions.

There is increasing evidence of sex differences in the epidemiology and pathophysiology of PDN. Indeed, males develop neuropathy earlier than females [10] and PDN is more frequent in men than in women [11, 12]. Also, nerve conduction abnormalities are more frequent and severe in males than in females [13, 14]. On the contrary, neuropathic pain and negative sensory symptoms are more frequent in female than in male patients [14]. Sex differences have been also reported in experimental models of PDN. For instance, the paw withdrawal threshold is reduced more in females than in males [15]. On the contrary, male exhibited greater intraepidermal nerve fiber density (IENF) loss than females [16].

Neuroactive steroids may be involved in the generation of sex differences in PDN. Neuroactive steroids are important physiological regulators of neural function and their levels are sex dimorphic both in the central and peripheral nervous system of control animals and also in nervous pathologies [17]. We previously reported that diabetes, in an advanced stage of the disease, influences local levels of neuroactive steroids in the PNS in a sex-dimorphic way [18]. Interestingly, also, their protective effects against alterations induced by diabetes show a sex-dimorphic feature [19, 20].

In this work, we have explored the possible existence of sex differences in the impact of early diabetes on the content and distribution of anterograde and retrograde motor proteins as well as on different mediators of the mitochondrial biogenesis and dynamics in the PNS.

## Methods

### Animals

Two-month-old male and female Sprague-Dawley rats (Crl:CD BR, Charles River, Lecco, Italy) were randomly assigned to control or diabetic groups. Diabetes was induced by a single intravenous injection of freshly prepared streptozotocin (60 mg/kg; Sigma, Italy) in citrate buffer 0.09 M pH 4.8 [18]. To determine the different phases of the estrous cycle, female rats were monitored by daily vaginal smears and only those demonstrating at least two consecutive 4-day cycles were used in the study. One month after the induction of diabetes rats were weighed, deeply anesthetized with isoflurane and sacrificed. Females were sacrificed on diestrus day. Sciatic nerve and dorsal root ganglia (DRG) were collected for biochemical analysis.

### Preparation of total DRG extract

After dissection, the DRG from each rat was homogenized in lysis buffer (PBS, pH 7.4, added with 1% Nonidet P-40) supplemented with protease and phosphatase inhibitor cocktails (Roche Diagnostic spa, Monza, Italy) with a TissueLyser II (Qiagen, Milano, Italy) instrument. The resulting homogenate was sonicated (17 kHz, 10 s each) and then centrifuged at 1200 g at 4 °C for 10 min. The supernatant was stored at − 80 °C until use.

### Axoplasm extraction

Axoplasm from rat sciatic nerve was extracted according to the method of Rishal and collaborators [21]. Briefly, after dissection, the epineurium was removed from the sciatic nerve and fascicles were then separated by mechanical dissociation with fine forceps. Fascicles were then incubated for 2 h in PBS × 0.2 with protease and phosphatase inhibitors at room temperature (RT). After three washes in the same buffer, axoplasm was extracted incubating the fascicles in PBS × 1 with protease and phosphatase inhibitors at RT for 30 min, followed by centrifugation at 10,000*g* for 10 min. The supernatant (axoplasm extract) was stored at − 80 °C until use.

### Western blot analysis

Equal amounts of protein were separated on SDS-PAGE (4–15% gradient gel; Bio-Rad, Milan, Italy), and Western blot was performed as described previously [22].

Incubation with specific antibodies (Table 1) was performed overnight at 4 °C. After washing for 1 h in PBS containing 0.1% Tween-20, the membranes were incubated with the proper secondary antibody for 2 h and visualized using enhanced chemiluminescence (ECL) method (Bio-Rad, Milan, Italy) according to the manufacturer’s instructions. Chemiluminescent signals were acquired with a ChemiDoc TM XRS+ system (Bio-Rad, Milan, Italy) and the digital quantification of immunoreactive bands was performed using Image Lab TM software version 3.0 (Bio-Rad, Milan, Italy). Then, concentration of each target protein was normalized versus GAPDH. When phosphorylation of DRP1 was measured, membranes were first probed for the phosphorylated form of the protein, then stripped, and probed for the total protein.

**Table 1:** List of primary antibodies

Primary antibody	Sample	Antibody dilution	Source
KIF5A	Total Extracts DRG	1:1.000	Proteintech (21186-1-AP)
	Isolated Axoplasm	1:500	
KIF5B	Total Extracts DRG	1:1.000	Proteintech (21632-1-AP)
	Isolated Axoplasm	1:500	
KIF1A	Total Extracts DRG	1:500	Abcam (ab180153)
	Isolated Axoplasm	1:200	
Myosin Va	Total Extracts DRG	1:500	Cell signaling (3402S)
	Isolated Axoplasm	1:200	
Dynein	Total Extracts DRG	1:500	Sigma (D5167)
	Isolated Axoplasm	1:200	
Mitoprofile total OXPHOS	Total Extracts DRG	1:1.000	Abcam (ab10413)
DRP1	Total Extracts DRG	1:500	Cell signaling (14647S)
DRP1-Ser616	Total Extracts DRG	1:500	Signalway Antibody (12749)
OPA1	Total Extracts DRG	1:500	Cell signaling (80471S)

### Quantitative real-time PCR (RT-qPCR)

RNA and DNA from snap-frozen DRG were extracted using the proper MiniPrep kit (Zymo Research, Irvine, CA, USA) following manufacturing protocol. RNA and DNA were quantified by Nano-DropTM 2000 (ThermoFisher scientific, Milano, Italy). TaqMan quantitative real-time PCR was performed by CFX96 real-time system (Bio-Rad Laboratories, Segrate, Italy). RNA samples were run in duplicate as multiplexed reactions with a normalizing internal control (18 s rRNA) (Life Technologies Monza, Italy). Mitochondrial DNA (mt-DNA) content was analyzed assessing mitochondrial cytochrome c oxidase subunit 2 (mt-CoxII) and 36B4 content as mitochondrial and nuclear encoded genes, respectively. For KIF1A (Rn01427419), the probe was purchased from Life Technologies (Monza, Italy). For the other motor proteins, specific TaqMan MGB probes and primers sequence (Table 2) were purchased from Eurofins MWG-Operon (Milano, Italy).

**Table 2:** Eurofins MWG-Operon primer sequences

Gene	Forward	Reverse
36B4	GGATGACTACCCAAAATGCTTC	TGGTGTTCTTGCCCATCAG
KIF5A	AGGTGCTGAATGGACTGATG	ACCTCTGACTTGATCTTGCTG
KIF5B	GAATCTGTGGACTCCCTTGG	GTTTCTCTGTGACTCTGGATCTG
Dync1i1	ACCCCTATGTCTCCCTCTTC	CTTCTCCCAAGTTCTGAGTCTG
MyoVa	GATAGAAGGAGTGGACGATGC	GAGACGCAAACCCAACATTG
PGC-1α	TGAGGAATGCACCGTAAATC	GTACAGCTCGAAGTCAGTTTC
mt-Cox II	ATTGTATTCCTCATCAGCTCCC	TGACAGCTGGGAGAATTGTTC

### Liquid chromatography tandem mass spectrometry analysis (LC-MS/MS)

Sciatic nerves were extracted and purified as previously described [23]. Quantitative analysis for neuroactive steroid levels was performed on the basis of calibration curves extracted and analyzed as the tissues samples. Positive atmospheric pressure chemical ionization (APCI+) experiments were performed using a linear ion trap-mass spectrometer (LTQ, ThermoElectron Co., San Jose, CA, USA) equipped with a Surveyor liquid chromatography (LC) Pump Plus (ThermoElectron Co., San Jose, CA, USA). The analytical conditions were previously described [24].

DRG from the different experimental groups were lysed in 250 μl methanol/acetonitrile 1:1 containing [U-13C6]-Glucose-1 ng/μl (internal standard, Sigma Aldrich, 389374) by tissue lyser and spun at 20,000 g for 5 min at 4 °C. Supernatant was then passed through a regenerated cellulose filter, dried and resuspended in 100 μl of MeOH for subsequent analysis. Data were obtained on an API-4000 triple quadrupole mass spectrometer (AB Sciex) coupled with an HPLC system (Agilent) and CTC PAL HTS autosampler (PAL System). The identity of ATP was confirmed using pure standard. Quantification of ATP was performed with a liquid chromatography/tandem mass spectrometry (LC-MS/MS) method using a cyano-phase LUNA column (50 mm × 4.6 mm, 5 μm; Phenomenex). Methanolic samples were analyzed by 8 min run in negative ion mode with a multiple reaction monitoring (MRM) transition. The mobile phases were phase A: 5 mM ammonium acetate pH 7.00 in water, and phase B: 5 mM ammonium acetate pH 7.00 in MeOH. The gradients were as follows: T_0_ 80%A; T_4min_ 0%A; T_5min_ 0%A; T_5.1min_ 80%A and T_8min_ 80%A with a flow rate of 500 μl/min. MultiQuant™ software (version 3.0.2) was used for data analysis and peak review of chromatograms. Quantitative evaluation of ATP was performed based on calibration curves with pure standard; then, data were normalized on protein content assessed by BCA method.

### Statistical analysis

Unpaired Student’s *t* test was applied to couples of independent variables. Data from experiments with more than two groups were analyzed by two-way analysis of variance (ANOVA), with sex and disease as two independent variables, followed by the Bonferroni post hoc test. All analyses were performed using GraphPad PRISM (version 5).

## Results

Table 3 reports body weight and glucose levels. As expected, both male and female diabetic rats had high blood glucose at the end of the experiment. On the contrary, only male animals had significantly less weight gain than non-diabetic controls while in female animals, administration of streptozotocin (STZ) did not significantly modify the body weight.

**Table 3:** Body weight and glucose levels

Animal	Body weight (g)	Body weight (g)	Blood glucose (mg/dl)
	Before STZ injection	At sacrifice	At sacrifice
M Ctrl	255.4 ± 2.5	405.0 ± 7.8	106 ± 3
M STZ	263.2 ± 4.1	344.0 ± 9.3***	530 ± 2***
F Ctrl	177.1 ± 2.9	230.1 ± 4.6	102 ± 4
F STZ	184.7 ± 1.8	223.5 ± 7.5	544.4***

Data are expressed as mean ± SEM (*n* = 6). Analysis by Student’s *t* test ***p < 0.001 vs. Ctrl

*M* male, *F* female, *Ctrl* control, *STZ* streptozotocin

### Axonal motor protein expression is altered by diabetes in a sex-dimorphic way

We analyzed mRNA levels and the protein content of molecular motors in the DRG homogenates and isolated axoplasm. This allowed us to evaluate the different distribution of motor proteins between the cell body compartment (DRG) and the axon compartment (axoplasm) and to avoid confounding results due to the ubiquitous expression of KIF5B, Dynein and Myosin Va [3, 25, 26].

### Short-term diabetes changes KIF5A protein levels in a sex-dimorphic way

**Fig. 1:**
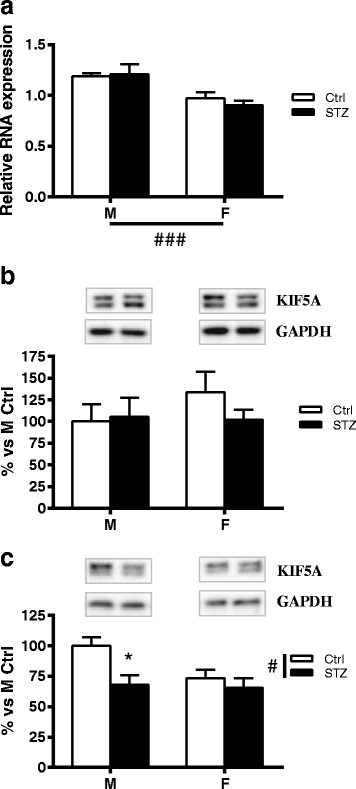
Effect of short-term diabetes on KIF5A. **a** RNA was extracted from DRG of control (Ctrl) and diabetic (streptozotocin, STZ) male (M) and female (F) rats. RT-PCR was used to analyze mRNA levels of KIF5A. **b** KIF5A protein levels analyzed by immunoblotting in DRG total extracts of Ctrl and STZ M and F rats. **c** KIF5A protein levels analyzed by immunoblotting in isolated axoplasm of Ctrl and STZ M and F rats. Representative Western blots are presented above the graphs, with the respective loading controls (GAPDH). The densitometry of each band was analyzed, and the results are expressed as percentage of M Ctrl. The columns represent the mean ± SEM, *n* = 6 animals for each group. The effects of sex, diabetes, and the interaction sex by diabetes were analyzed using two-way ANOVA (significance: #*p* < 0.05; ###*p* < 0.001) followed by Bonferroni post hoc test (significance: *p < 0.05)

Two-way ANOVA revealed a significant effect of sex (*p* < 0.001) on KIF5A mRNA expression in DRG (Fig. 1a). On the contrary, no significant effects of sex or diabetic status were observed in DRG protein levels (Fig. 1b). The analysis of isolated axoplasm revealed a significant effect of diabetes (two-way ANOVA *p* < 0.05). In particular, post hoc analysis indicated that KIF5A protein levels were significantly decreased in male diabetic rats compared to control males (*p* < 0.05) but not in female animals (Fig. 1c).

### Short-term diabetes changes KIF5B mRNA expression and protein levels in a sex-dimorphic way

**Fig. 2:**
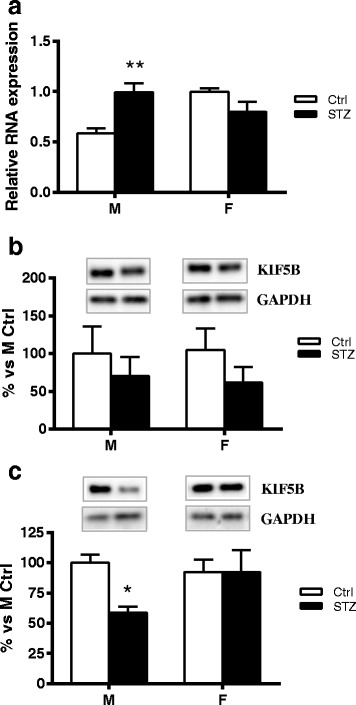
Effect of short-term diabetes on KIF5B. **a** RNA was extracted from DRG of control (Ctrl) and diabetic (streptozotocin, STZ) male (M) and female (F) rats. RT-PCR was used to analyze mRNA levels of KIF5B. **b** KIF5B protein levels analyzed by immunoblotting in DRG total extracts of Ctrl and STZ M and F rats. **c** KIF5B protein levels analyzed by immunoblotting in isolated axoplasm of Ctrl and STZ M and F rats. Representative Western blots are presented above the graphs, with the respective loading controls (GAPDH). The densitometry of each band was analyzed and the results are expressed as percentage of M Ctrl. The columns represent the mean ± SEM, *n* = 6 animals for each group. The effects of sex, diabetes, and the interaction sex by diabetes were analyzed using two-way ANOVA followed by Bonferroni post hoc test (significance: *p < 0.05; **p < 0.01)

Two-way ANOVA analysis showed a very significant interaction between sex and diabetic status on KIF5B mRNA expression in DRG (*p* < 0.001). In agreement with previous observations obtained in male hippocampus [6, 9], diabetes induced a significant increase of KIF5B mRNA expression in male DRG (Bonferroni post hoc analysis, *p* < 0.01). However, no significant changes of KIF5B mRNA expression were observed in DRG of female diabetic rats compared to control (Fig. 2a). No differences were observed between control or diabetic males and females, for KIF5B protein levels in DRG homogenates (Fig. 2b). However, in agreement with previous observations [6], and similarly to what observed for KIF5A, Western blot analysis of axoplasm preparation revealed a significant decrease in KIF5B protein levels (Bonferroni post hoc analysis, *p* < 0.05) in male diabetic rats compared to control males. In contrast, no significant changes were observed in females (Fig. 2c). Two-way ANOVA analysis showed no significant effect of sex or diabetic status on KIF5B protein levels.

### Short-term diabetes changes KIF1A mRNA expression and protein levels in a sex-dimorphic way

**Fig. 3:**
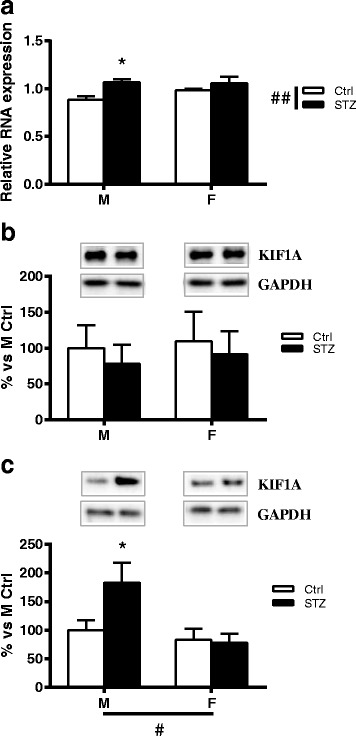
Effect of short-term diabetes on KIF1A. **a** RNA was extracted from DRG of control (Ctrl) and diabetic (streptozotocin, STZ) male (M) and female (F) rats. RT-PCR was used to analyze mRNA levels of KIF1A. **b** KIF1A protein levels analyzed by immunoblotting in DRG total extracts of Ctrl and STZ M and F rats. **c** KIF1A protein levels analyzed by immunoblotting in isolated axoplasm of Ctrl and STZ M and F rats. Representative Western blots are presented above the graphs, with the respective loading controls (GAPDH). The densitometry of each band was analyzed, and the results are expressed as percentage of M Ctrl. The columns represent the mean ± SEM, *n* = 6 animals for each group. The effects of sex, diabetes, and the interaction sex by diabetes were analyzed using two-way ANOVA (significance: #*p* < 0.05; ##*p* < 0.01) followed by Bonferroni post hoc test (significance: *p < 0.05)

Two-way ANOVA analysis revealed a significant effect of diabetes (*p* < 0.01) on KIF1A mRNA levels in DRG. In particular, and in agreement with previous findings [6], diabetes induced a significant increase of KIF1A mRNA levels in males (Bonferroni post hoc analysis, *p* < 0.05). However, this did not occur in female animals (Fig. 3a). Nevertheless, no significant changes were detected between control or diabetic males and females, for the KIF1A protein levels in DRG (Fig. 3b). The analysis of isolated axoplasm revealed a significant effect of sex (two-way ANOVA *p* < 0.05). In addition, post hoc analysis indicated that, similarly to what previously observed by others in axons of hippocampal neuron [6], KIF1A protein levels were significantly increased in male diabetic rats compared to control males (*p* < 0.05). In contrast, no significant changes were observed in female diabetic rats compared to control females (Fig. 3c).

### Short-term diabetes changes Myosin Va protein levels in a sex-dimorphic way

**Fig. 4:**
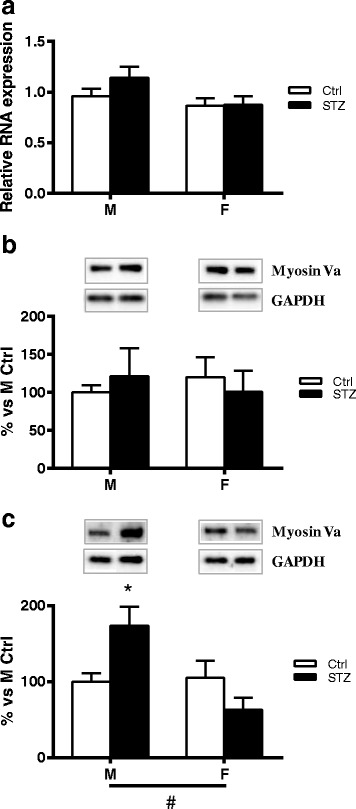
Effect of short-term diabetes on Myosin Va. **a** RNA was extracted from DRG of control (Ctrl) and diabetic (streptozotocin, STZ) male (M) and female (F) rats. RT-PCR was used to analyze mRNA levels of Myosin Va. **b** Myosin Va protein levels analyzed by immunoblotting in DRG total extracts of Ctrl and STZ M and F rats. **c** Myosin Va protein levels analyzed by immunoblotting in isolated axoplasm of Ctrl and STZ M and F rats. Representative Western blots are presented above the graphs, with the respective loading controls (GAPDH). The densitometry of each band was analyzed, and the results are expressed as percentage of M Ctrl. The columns represent the mean ± SEM, *n* = 6 animals for each group. The effects of sex, diabetes, and the interaction sex by diabetes were analyzed using two-way ANOVA (significance: #*p* < 0.05) followed by Bonferroni post hoc test (significance: *p < 0.05)

Two-way ANOVA showed no significant effect of sex or diabetic status on Myosin Va mRNA levels as well as protein levels in DRG (Fig. 4a, b). However, Western blot analysis of axoplasm preparation revealed a significant effect of sex (two-way ANOVA *p* < 0.05) and a significant interaction between sex and diabetic status (two-way ANOVA *p* < 0.05). In addition, post hoc analysis indicated that Myosin Va levels were significantly higher in the axoplasm of diabetic males than in control males (*p* < 0.05). As observed for the others motor proteins analyzed, no significant changes were observed between diabetic and control female rats (Fig. 4c).

### Short-term diabetes does not affect Dynein mRNA expression and protein levels

**Fig. 5:**
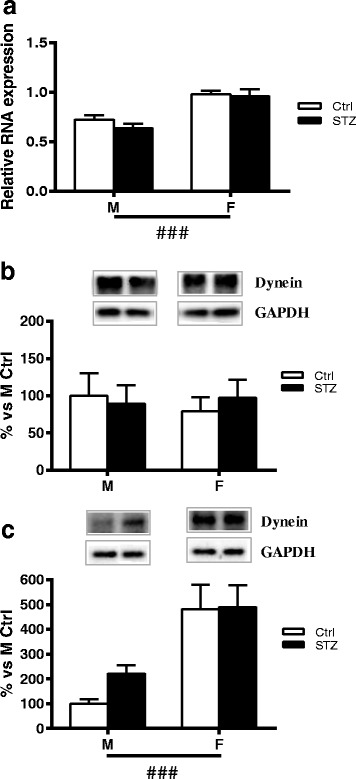
Effect of short-term diabetes on Dynein. **a** RNA was extracted from DRG of control (Ctrl) and diabetic (streptozotocin, STZ) male (M) and female (F) rats. RT-PCR was used to analyze mRNA levels of Dynein. **b** Dynein protein levels analyzed by immunoblotting in DRG total extracts of Ctrl and STZ M and F rats. **c** Dynein protein levels analyzed by immunoblotting in isolated axoplasm of Ctrl and STZ M and F rats. Representative Western blots are presented above the graphs, with the respective loading controls (GAPDH). The densitometry of each band was analyzed, and the results are expressed as percentage of M Ctrl. The columns represent the mean ± SEM, *n* = 6 animals for each group. The effects of sex, diabetes, and the interaction sex by diabetes were analyzed using two-way ANOVA (significance: ###*p* < 0.001) followed by Bonferroni post hoc test

In agreement with previous findings in hippocampus [6], diabetes did not change Dynein mRNA expression and protein levels in DRG. Two-way ANOVA, however, showed a significant effect of sex on Dynein mRNA levels (*p* < 0.001) (Fig. 5a, b). Western blot analysis of axoplasm preparation also revealed a significant effect of sex (two-way ANOVA *p* < 0.001). No difference was observed, however, between control or diabetic males and females for this molecular motor (Fig. 5c).

### Mitochondrial biogenesis is altered by diabetes in a sex-dimorphic way

Following previous studies suggesting that mitochondrial function and dynamic is impaired in diabetic neuropathy [2], we examined mitochondrial biogenesis in DRG of control and diabetic male and female rats.

### Short-term diabetes alters PGC-1α gene expression in a sex-dimorphic way

**Fig. 6:**
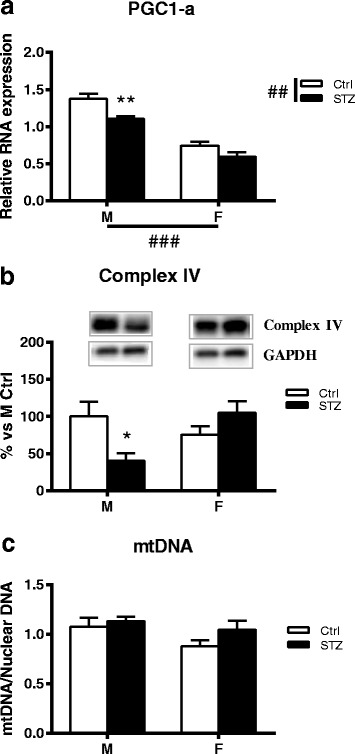
Effect of short-term diabetes on PGC-1α gene expression, respiratory chain complex IV expression and mitochondrial DNA. **a** RNA was extracted from DRG of control (Ctrl) and diabetic (streptozotocin, STZ) male (M) and female (F) rats. RT-PCR was used to analyze mRNA levels of PGC1-α. **b** Immunoblot of mitochondrial OXPHOS Complex IV from DRG total extracts of Ctrl and STZ M and F rats. Representative Western blots are presented above the graph, with the respective loading control (GAPDH). The densitometry of each band was analyzed, and the results are expressed as percentage of M Ctrl. **c** Total DNA was isolated from non-diabetic Ctrl and STZ rats and run for quantitative real-time PCR to obtain a relative ratio of mt-CoxII (a gene coded on the mitochondrial genome) over 36B4 (a gene coded on nuclear genome), as indicator for relative mt-DNA copy number. The columns represent the mean ± SEM, *n* = 6 animals for each group. The effects of sex, diabetes, and the interaction sexby diabetes were analyzed using two-way ANOVA (significance: ##*p* < 0.01; ###*p* < 0.001) followed by Bonferroni post hoc test (significance: *p < 0.05; **p < 0.01)

Real-time PCR analysis of PGC-1α revealed a very significant effect of sex (two-way ANOVA *p* < 0.001) and a very significant effect of diabetic status (two-way ANOVA *p* < 0.01) on PGC-1α mRNA levels. In particular, and in agreement with the findings of Choi and collaborators [27], 1 month of diabetes induced a significant decrease of PGC-1α mRNA levels in males (Bonferroni post hoc analysis, *p* < 0.01). On the contrary, diabetic status did not affect this parameter in female rats (Fig. 6a).

To determine whether the observed decreased expression of this transcriptional coactivator was associated with altered mitochondrial biogenesis and function, we measured the protein content of different subunits of the respiratory chain complexes (i.e., complex I–II–III–IV) and the content of mtDNA.

### Short-term diabetes alters the expression of respiratory chain complex IV and total ATP content in a sex-dimorphic way

As reported in Fig. 6b, quantitative Western blot showed a significant decrease of respiratory chain complex IV in DRG of short-term diabetic male rats (Bonferroni post hoc analysis, *p* < 0.05). On the contrary, in female animals, diabetes did not induce a significant change in this parameter. Two-way ANOVA analysis showed a significant interaction between sex and diabetic status on respiratory chain complex IV expression (*p* < 0.05). No significant differences of complex I, II, III, and V were observed among the four groups (data not shown).

In addition, by LC-MS/MS analysis, we assessed total ATP levels. The results showed a decrease of ATP levels in male STZ DRG compared to control animals, while no changes were observed in female animals [M ctrl (*n* = 7) 0.37 ± 0.04; M STZ (*n* = 7) 0.19 ± 0.02 (Bonferroni post hoc analysis, *p* < 0.001); F ctrl (*n* = 7) 0.26 ± 0.03; F STZ (*n* = 7) 0.24 ± 0.01. Data are expressed as ng/μg of protein ± SEM].

### Short-term diabetes does not alter mitochondrial DNA

To determine if a change in mitochondrial DNA (mtDNA) occurred in DRG of diabetic male and female rats, mtDNA copy number was measured using quantitative real-time PCR to obtain a relative ratio of mtDNA over nuclear DNA (nDNA). The ratio of mtDNA to nDNA was similar in control male and female rats as well as in diabetic rats (Fig. 6c).

### Short-term diabetes activates Drp1 activity in a sex-dimorphic way

To further understand the effect of diabetic status on mitochondrial dynamics, we examined the expression of proteins known to regulate mitochondrial fission/fusion (MFN2, OPA1, DRP1) in DRG of control and diabetic male and female rats.

First, we assessed whether diabetic status changes DRP1 phosphorylation at serine 616 (DRP1P). This post-translational modification activates DRP1 and causes DRP1 to translocate to the mitochondria and form multimers that circumferentially constrict the mitochondrion and initiate fission [28].

As expected and in line with previous findings [29], 1 month of diabetes did not significantly modify the protein levels of total DRP1 in either male or female rats (data not shown).

**Fig. 7:**
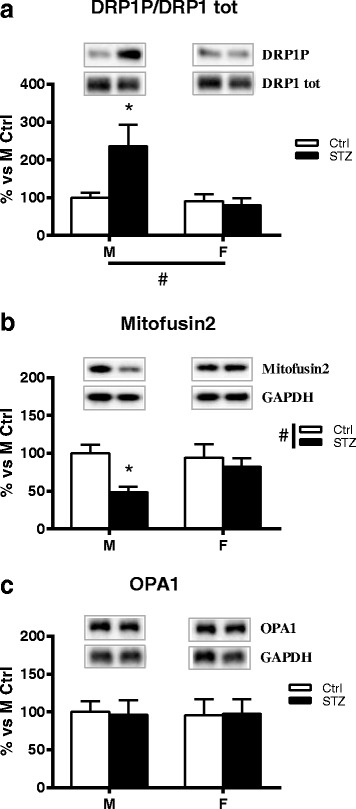
Effect of short-term diabetes on proteins involved in mitochondrial fission/fusion: MFN2, Drp1 and OPA1. **a** Immunoblot of DRP1 phosphorylation from DRG total extracts of control (Ctrl) and diabetic (streptozotocin, STZ) male (M) and female (F) rats. The bar graph represents densitometric analysis and statistical evaluation of DRP1 phosphorylation over total DRP1 (DRP1P/DRP1 tot); representative western blot of phosphorylated and total DRP1 is presented above the graph. **b** Immunoblot of MFN2 from DRG total extracts of Ctrl and STZ M and F rats. **c** Immunoblot of OPA1 from DRG total extracts of Ctrl and STZ M and F rats. Representative Western blots are presented above the graphs, with the respective loading controls (GAPDH). The densitometry of each band was analyzed and the results are expressed as percentage of M Ctrl. The columns represent the mean ± SEM, *n* = 6 animals for each group. The effects of sex, diabetes, and the interaction sex by diabetes were analyzed using two-way ANOVA (significance: #*p* < 0.05) followed by Bonferroni post hoc test (significance: *p < 0.05)

However, two-way ANOVA analysis showed a significant effect of sex on DRP1P. In particular, we detected a significant increase in the expression of activated DRP1 in the DRG of diabetic males when compared with control males (Bonferroni post hoc analysis, *p* < 0.05) whereas no significant changes were observed between diabetic and control female rats (Fig. 7a).

### Short-term diabetes alters the expression of Mfn2 in a sex-dimorphic way

Mitofusin 2 (MFN2) is an outer mitochondrial membrane protein that regulates mitochondrial fusion and is also a key component in the regulation of mitochondrial transport [30]. As shown in Fig. 7b, two-way ANOVA revealed a significant effect of diabetes (*p* < 0.05) on MFN2 levels. In particular, post hoc analysis indicated that diabetes significantly decreased MFN2 protein levels in male rats (Bonferroni post hoc analysis, *p* < 0.05). On the contrary, diabetes did not induce a significant change in this parameter in female animals (Fig. 7b).

### Short-term diabetes does not alter the expression of OPA1

Optic Atrophy Protein 1 (Opa1) is one of the key players of the fusion process. It is localized in the inner mitochondrial membrane, interacting with both MFN1 and 2. Two-way ANOVA analysis showed no significant effect of diabetes or sex on OPA1 expression (Fig. 7c).

### Neuroactive steroid levels are altered by diabetes in a sex-dimorphic way

The above results showed a clear sex difference in the impact of short-term diabetes in the content of motor proteins and mitochondrial function. These observations pointed to a possible role of neuroactive steroids. On this basis, we explored whether neuroactive steroid levels in the sciatic nerve of control and diabetic male and female rats were differently affected by short-term diabetes.

We measured the levels of progesterone and its metabolites (dihydroprogesterone, allopregnanolone, and isopregnanolone) and testosterone and its metabolites (dihydrotestosterone, 5α-androstane-3α,17β-diol and 5α-androstane-3β,17β-diol) as well as 17α-estradiol and 17β-estradiol by LC-MS/MS in the sciatic nerve. The results are shown in Table 4. As expected, levels of progesterone and its metabolites were higher in females than in males whereas the levels of testosterone and its active metabolite dihydrotestosterone were higher in male than in female animals. In males, diabetes induced a very significant decrease in the levels of testosterone and dihydrotestosterone and a significant increase in the levels of allopregnanolone. On the contrary, none of the neuroactive steroids analyzed showed alterations in its levels in female diabetic animals.

**Table 4:** Neuroactive steroids in the sciatic nerve of male and female control and diabetics

	Male		Female	
	Ctrl	STZ	Ctrl	STZ
Progesterone	3.17 ± 0.3	3.21 ± 0.5	5.68 ± 0.7	4.00 ± 0.3
Dihydroprogesterone	1.15 ± 0.5	1.92 ± 0.7	17.60 ± 3.6	16.30 ± 3.2
Allopregnanolone	3.28 ± 0.2	7.63 ± 1.7***	10.50 ± 0.9	10.70 ± 1.4
Isopregnanolone	0.37 ± 0.1	0.27 ± 0.1	16.40 ± 2.2	16.80 ± 4.1
Testosterone	2.59 ± 0.6	0.55 ± 0.4***	0.42 ± 0.1	0.35 ± 0.1
Dihydrotesterone	12.10 ± 0.8	2.23 ± 0.6***	UDL	UDL
5α-Androstane-3α 17β-diol	0.95 ± 0.5	0.31 ± 0.2	0.66 ± 0.3	0.10 ± 0.1
5α-Androstane-3β 17β-diol	5.40 ± 1.3	4.50 ± 0.6	4.21 ± 1.0	5.92 ± 0.6
17α -Estradiol	0.56 ± 0.1	0.63 ± 0.1	0.67 ± 0.1	0.74 ± 0.1
17β -Estradiol	0.43 ± 0.1	0.44 ± 0.1	0.78 ± 0.3	0.60 ± 0.7

Neuroactive steroid levels were evaluated by liquid chromatography tandem mass spectrometry. Data are expressed as pg/mg of tissue ± SEM. The number of animals utilized was 6. Analysis by Student’s *t* test ***p < 0.001 vs Ctrl

*Ctrl* control, *STZ* diabetics, *UDL* under detection limit

## Discussion

Our findings indicate that short-term diabetes alters the expression of genes involved in mitochondrial function and dynamics as well as the content of key motor proteins important for axonal transport in the PNS of males but not in females. Moreover, we showed that early diabetes is associated with a decrease in the levels of specific neuroactive steroids in males but not in females, suggesting that the increased resistance of females to diabetes-induced axonal transport damage may be related to the positive effect of female sex steroids on mitochondria.

Specifically, we showed that short-term diabetes alters both the mRNA levels and axoplasm content of KIF1A and KIF5B and axoplasm content of KIF5A and Myosin Va, but only in male animals. No changes in dynein levels were detected. Since dynein is involved in retrograde transport, this finding suggests that the anterograde but not the retrograde transport is impaired in an early stage of the disease in male animals.

KIF5A is known to be responsible for the transport of neurofilaments (NF) [31]. NF are affected since the earliest stage of the disease contributing to the axonal degeneration [32, 33]. The decrease in the expression of KIF5A in the axoplasm of males may be due to the imbalance in protein synthesis or axonal transport deficit. Protein and mRNA levels of KIF5A were not affected by diabetes in the DRG, suggesting that the local depletion of KIF5A in the axoplasm is not a consequence of a change at the transcript level but it might be the result of a defect in the molecular motor function slowing down KIF5A mobility along the axon.

In addition to KIF5A, KIF5B protein levels were also decreased in the isolated axoplasm in males but not in females. As for KIF5A, no changes were observed for KIF5B protein levels in the DRG. This result suggests that also in this case, the decrease of KIF5B might result from a defect in the molecular motor function. This observation is consistent with what observed by Baptista and collaborators [6] who showed that KIF5B protein immunoreactivity decreased in the axons of hippocampal cell cultures treated for 7 days with glucose. A previous study in STZ-induced diabetic rats, however, reported an increase in KIF5B protein content in the sciatic nerve of diabetic animals [9]. A possible explanation for this discrepancy could be the strategy used to measure protein levels. In the previous study, levels of KIF5B were determined in total sciatic homogenate using ELISA method. It is worth remembering that KIF5B is expressed ubiquitously [34]; thus, measuring KIF5B levels in a total sciatic nerve homogenate may not provide accurate estimates of this motor protein in axons. To avoid confounding results due to the presence of this protein in Schwann cells [35], we performed our analysis in the isolated axoplasm.

Besides the decrease of KIF5B protein levels in the axoplasm, we found a significant increase in its mRNA levels in the DRG of diabetic male animals when compared to controls. An overexpression of KIF5B was also found in the hippocampal at 2 weeks of diabetes [6]. We can speculate that this increased expression might function as a compensatory mechanism to re-establish the decrease level of this motor along the axon. Further analysis, however, will be necessary to support this hypothesis.

Another important molecular motor essential for the sensory neuronal function is KIF1A. Interestingly, recent evidence showed that KIF1A, besides its known role in transporting synaptic vesicles [36], is also involved in the transport of the NGF receptor TrkA in axons of DRG neurons. This transport plays an important role in PI3K-mediated cell survival and sensitization supporting neuronal viability [37]. Our findings indicate that KIF1A mRNA levels are significantly increased in DRG of diabetic males when compared to controls. In addition, a significant increase of this motor protein was evident in the axoplasm of male diabetic animals. Due to the role of KIF1A in mediating the signaling pathway responsible for sensory function in the PNS [37], it is tempting to speculate that sex-specific alteration of this motor protein found in diabetic animals may ultimately account for sexual dimorphism in pain and analgesia observed in diabetic humans and in diabetes animal models [14, 15].

Diabetes also induced an alteration in Myosin Va in males but not in females. Myosin Va protein levels were significantly higher in the axoplasm of male diabetic animals compared to male controls whereas no changes were observed either in the protein or in the mRNA levels in the DRG. Myosin Va has been implicated in a wide range of cargo transport, supporting multiple functions within neurons [4]. Previous studies showed a decrease in myosin Va in brain neuronal cell bodies of STZ-rats at an early stage of diabetes, as well as in jejunal musculomotor nerve terminals 16 weeks after induction of diabetes [38, 39]. These finding are in contrast with our results. One possibility for this discrepancy may be that diabetes affects the expression and content of this motor protein in a time- and tissue-specific way. Importantly, myosin Va can function as a tether for kinesins preventing their diffusion away from the microtubule track, enhancing kinesin processivity [40]. Thus, the increase of myosin Va at an early stage of diabetes might be interpreted as an attempt to cope with the diabetes-mediated impairment of axonal transport. Indeed, it has been demonstrated that myosin can be locally synthesized in the sciatic nerve following nerve injury [25].

Axonal transport consumes high ATP levels through translocation of motor proteins [41–43]. Mitochondria are the site of oxidative phosphorylations (OXPHOS/respiratory chain complex I–V) where most of the energy is produced in the form of ATP derivatives. Previous studies have shown a reduced activity and protein expression of the mitochondrial respiratory chain in the DRG of rodents with type 1 and type 2 diabetes [44–46]. Furthermore, levels of the respiratory chain complex IV was found to be significantly reduced in IENF and subpapillary dermal fibers in skin samples of persons with diabetic neuropathy already at an early stage of the disease, prior to significant fiber loss [47]. In agreement with these previous reports, we detected a significant decrease in the expression of complex IV in the DRG of male diabetic animals. Importantly, this alteration was not detected in female rats, indicating that mitochondrial respiratory chain from female rats is not affected by diabetes in an early stage of the disease. This feature could be related to the sex differences in mitochondrial respiratory function. Indeed, female mitochondria have higher electron transport chain activity and ATP production [48, 49]. Moreover, as observed in the brain of young mice, females have higher NADH-linked respiration than males [50]. Thus, it is reasonable to suppose that the greater functional capacities in female mitochondria might account for the higher resistance to diabetic insults observed in our study.

PGC-1α is a transcriptional coactivator that co-ordinates the expression of multiple mitochondrial proteins involved in mitochondrial biogenesis. It also regulates the expression of respiratory chain complex I to V [51]. The analysis of the expression of PGC-1α in DRG revealed a significant effect of sex with an overall lower level of PGC-1α transcript in female rats. This is in agreement with previous findings in the mouse brain [52]. We also found a significant effect of diabetes. Interestingly, PCG-1α mRNA levels were decreased in diabetic males compared with control males. On the contrary, this effect of diabetes was not detected in females. A decrease in PGC-1α expression was previously observed in DRG of rats and mice with type 1 or type 2 diabetes [53]. Moreover, in diabetic mice, knocking down PGC-1α worsens diabetic neuropathy [27]. In the light of our data, we propose that the downregulated expression of PGC-1α in diabetic males may be involved in the decrease in the levels of the respiratory chain complex IV found in the same animals. In agreement with the observations obtained in several cell lines by Li and colleagues [54], we observed that in male STZ DRG the decrease in the levels of complex IV secondarily results in a decline of ATP levels. These effects did not occur in female animals, suggesting that short-term diabetes alters, only in male animals, the mitochondrial function.

There is a complex relationship between bioenergetics and mitochondrial dynamics. Mitochondrial dynamics is a process used by these organelles to adapt their shapes, through frequent fusion and fission events, in response to changes in energy demand and supply [55]. Vincent and collaborators suggested that mitochondrial fission is increased in diabetes to meet the greater energetic requirements of a hyperglycemic state [56]. However, since fission does not require replication of mtDNA, rapid mitogenesis eventually leads to unhealthy mitochondria [56]. Alternatively, mitochondria fusion can be activated in response to mitochondrial stress to dilute mutated mtDNA and rescue damaged mitochondria via the acquisition of key components from healthy mitochondria [57]. One of the key regulators of mitochondrial fission is dynamin-1-like protein (DRP1). Inhibition of DRP1 results in decreased susceptibility to hyperglycemic damage in DRG neurons [29]. In the present study, we found a significant increase in the expression of activated DRP1 in the DRG of diabetic males, suggesting an overactivity of mitochondrial fission at short-term diabetes. In addition, the stable mtDNA expression observed here between control and diabetic animals indicates that fission of preexisting mitochondria is taking place with no alterations on mtDNA content, promoting mitochondrial fragmentation and probably impairing mitochondrial function. On the other hand, consistently with the results obtained here for other parameters, diabetes did not induce significant changes in the expression of DRP1 in females, indicating that also for this parameter, females are protected from diabetes-induced alterations.

Mitofusin 1 and 2 (MFN1 and MFN2) and optic atrophy protein 1 (OPA1) have a central role in mitochondrial fusion in both rodents and humans [58]. In our study, we did not find significant changes in OPA1 protein levels in DRG of diabetic animals. However, the expression of MFN2 was decreased in the DRG of male diabetic animals compared to control males. Again, no significant changes were observed in female animals. Previous works showed that MFN2 expression is reduced in patients with obesity or type 2 diabetes as well as in obese Zucker rats, a model of type 2 diabetes, suggesting that mitochondrial fusion is an important factor in the pathophysiology of these disorders [59, 60]. Here, we extended these observations reporting an impairment of this protein in a model of type 1 diabetes.

Interestingly, MFN2 also facilitates the axonal transport of mitochondria binding to kinesin motor proteins via the adaptor proteins Miro/Milton [30, 61]. Indeed, MFN2 conditional knockout mice display fragmented and aggregated mitochondria in dendrites [62]. Finally, this protein has an essential role in oxidative phosphorylation during mitochondrial respiration [63]. Thus, we can expect that the decrease of this key regulatory protein can worsen the neurodegenerative process acting at different levels: energetic, structural, and functional.

Mitochondrial function and biogenesis are regulated by sex hormones [64]. For example, estradiol increases the expression of PGC-1α in mouse female hearts [65] and enhances the activity of complex IV in rat brain mitochondria [66]. Estradiol and progesterone are also able to modulate mitochondrial fusion and fission genes, stimulating MFN1 and MFN2 expression [67]. We previously demonstrated in a model of long-term diabetes, that the levels of neuroactive steroids in PNS are differently affected by the disease in male and female animals [18]. Therefore, different levels of neuroactive steroids may contribute to sex differences in mitochondrial function of diabetic rats.

On this basis, we evaluated whether short-term diabetes changes the levels of testosterone and progesterone as well as their metabolites. We found that, in the sciatic nerve of male rats, short-term diabetes is associated with a decrease of testosterone and dihydrotestosterone. Interestingly, low levels of testosterone in men are associated with decreased expression of genes involved with energy metabolism and with reduced expression and activity of mitochondrial respiratory chain [68, 69]. Thus, it could be hypothesized that, at least in sciatic nerve, androgens may exert a positive effect on mitochondrial function and biogenesis in males. This effect seems not to be related to changes in androgen receptor, which is expressed in DRG [70]. Indeed, AR gene expression in DRG male rats is not affected by short-term diabetes (data not shown). Besides the decrease of these androgens, we also found an increase in allopregnanolone. A previous study demonstrated a protective role of this steroid in apoptosis-induced mitochondria release of cytocrome c [71]. We can speculate that the increase in allopregnanolone might be an attempt to cope with the diabetes-mediated impairment of mitochondria. It is worth noticing, however, that this increase is transient since the level of this steroid returns to control values at 3 months of diabetes [18]. On the other hand, no changes were observed between diabetic and control female rats. The positive effect of estrogens on mitochondrial functional capacities together with the stable levels of this steroid in diabetic female rats may explain in part why female mitochondria resulted protected from diabetic insult. Levels of estrogens, however, were not affected by diabetes also in males. Nevertheless, it is worth considering that estrogens can affect mitochondrial dynamics in sex-specific way, resulting in protection for female but not male mitochondria [67, 72].

## Conclusions

Our findings demonstrate that short-term diabetes differentially affects in males and females the levels of specific neuroactive steroids in the sciatic nerve. In addition, female mitochondria in the DRG were protected from diabetes whereas in the male DRG diabetes induced several alterations in the expression of proteins involved in mitochondrial function and dynamics. Finally, the levels of molecules involved in anterograde axonal transport were differentially affected by diabetes in the sciatic nerve of male and female rats. The preservation in the levels of neuroactive steroids in diabetic females may contribute to prevent mitochondrial alterations in these animals. In turn, proper functionality of mitochondria may preserve axonal transport in female diabetic rats.

## Abbreviations

Ctrl: Control
DRG: Dorsal root ganglia
DRP1: Dynamin-1-like protein
F: Female
IENF: Intraepidermal nerve fiber density
KIF: Kinesin family
LC-MS/MS: Liquid chromatography tandem mass spectrometry analysis
M: Male
MFN2: Mitofusin 2
mt-CoxII: Mitochondrial cytochrome c oxidase subunit 2
mt-DNA: Mitochondrial DNA
nDNA: Nuclear DNA
NF: Neurofilaments
Opa1: Optic Atrophy Protein 1
PDN: Peripheral diabetic neuropathy
PGC-1α: Peroxisome proliferator-activated receptor γ co-activator-1α
RT: Room temperature
STZ: Streptozotocin
